# Supplementary material for: A hierarchy of causes of death in senescent C. elegans
Source: bioRxiv. 2025 Aug 25:2025.08.21.671442. Preprint. [Version 1] doi: 10.1101/2025.08.21.671442 (PMC12407732; doi:10.1101/2025.08.21.671442)
Supplement: Supplement 2 [file NIHPP2025.08.21.671442v1-supplement-2.pdf]

bioRxiv preprint doi: https://doi.org/10.1101/2025.08.21.671442; this version posted August 25, 2025. The copyright holder for this preprint (which was not certified by peer review) is the author/funder, who has granted bioRxiv a license to display the preprint in perpetuity. It is made available under aCC-BY 4.0 International license.

Supplementary Information

A hierarchy of causes of death in senescent *C. elegans*

Contents Summary

Supplementary Figure 1. Effects of FUDR on senescent pathologies in *C. elegans* hermaphrodites (20˚C).

Supplementary Figure 2. Effects of *glp-4(bn2)* on aging pathologies (25˚C from L4).

Supplementary Figure 3. Effects of FUDR on lifespan and uterine tumor development (25˚C).

Supplementary Figure 4. Effects of FUDR on tumors in *C. tropicalis* and *P. pacificus*.

Supplementary Figure 5. Effects of treatment combinations to remove life-limiting pathologies.

Supplementary Figure 6. Carb suppresses enhancement of *daf-2* longevity by *glp-4(bn2)*.

Supplementary Table 1. Effects of 50 mM FUDR on lifespan in the presence of carbenicillin or kanamycin (20˚C).

Supplementary Table 2. Effects of *glp-4(bn2)* on lifespan in the presence of carbenicillin or kanamycin (25˚C from L4 stage).

Supplementary Table 3. Effects of *daf-16* on *glp-4* longevity (25˚C from L4).

Supplementary Table 4. Effects of *daf-12* on *glp-4* longevity (25˚C from L4).

Supplementary Table 5. Effects of FUDR on *glp-4(bn2)* lifespan (25˚C from L4 stage).

Supplementary Table 6. Verification of effects of FUDR on *glp-4(bn2)* lifespan (25˚C from L4 stage).

Supplementary Table 7. Effects of 50 mM FUDR on lifespan of N2 hermaphrodites and males in monoxenic liquid culture (20˚C, monoxenic liquid culture).

Supplementary Table 8. Effects of FUDR on lifespan when administered only after tumor development.

Supplementary Table 9. Effects of FUDR on lifespan in hermaphrodites and females of different free-living nematode species (*Caenorhabditis* species).

Supplementary Table 10. Effects of FUDR on lifespan in hermaphrodites and females of different free-living nematode species (*Pristionchus* species).

Supplementary Table 11. Effects of *vit-5,-6* RNAi, Kan and 50 μM FUDR on senescent pathologies (statistical comparisons).

Supplementary Table 12. Combined effects on lifespan of blocking infection, tumor development and vitellogenesis (20˚C).

bioRxiv preprint doi: https://doi.org/10.1101/2025.08.21.671442; this version posted August 25, 2025. The copyright holder for this preprint (which was not certified by peer review) is the author/funder, who has granted bioRxiv a license to display the preprint in perpetuity. It is made available under aCC-BY 4.0 International license.

Supplementary Table 13. Prevention of *E. coli* infection causes *ced-3* to shorten lifespan.

Supplementary Table 14. Carb suppresses enhancement of *daf-2* longevity by *glp-4(bn2)* (25˚C from L4 stage).

Supplementary Table 15. Mortality deconvolution analysis of effects of *glp-4(bn2)* on lifespan in *daf-2(m577)* (no Carb).

Other supplementary files

Supplementary Dataset 1. All raw lifespan data.

bioRxiv preprint doi: https://doi.org/10.1101/2025.08.21.671442; this version posted August 25, 2025. The copyright holder for this preprint (which was not certified by peer review) is the author/funder, who has granted bioRxiv a license to display the preprint in perpetuity. It is made available under aCC-BY 4.0 International license.

Supplementary figures

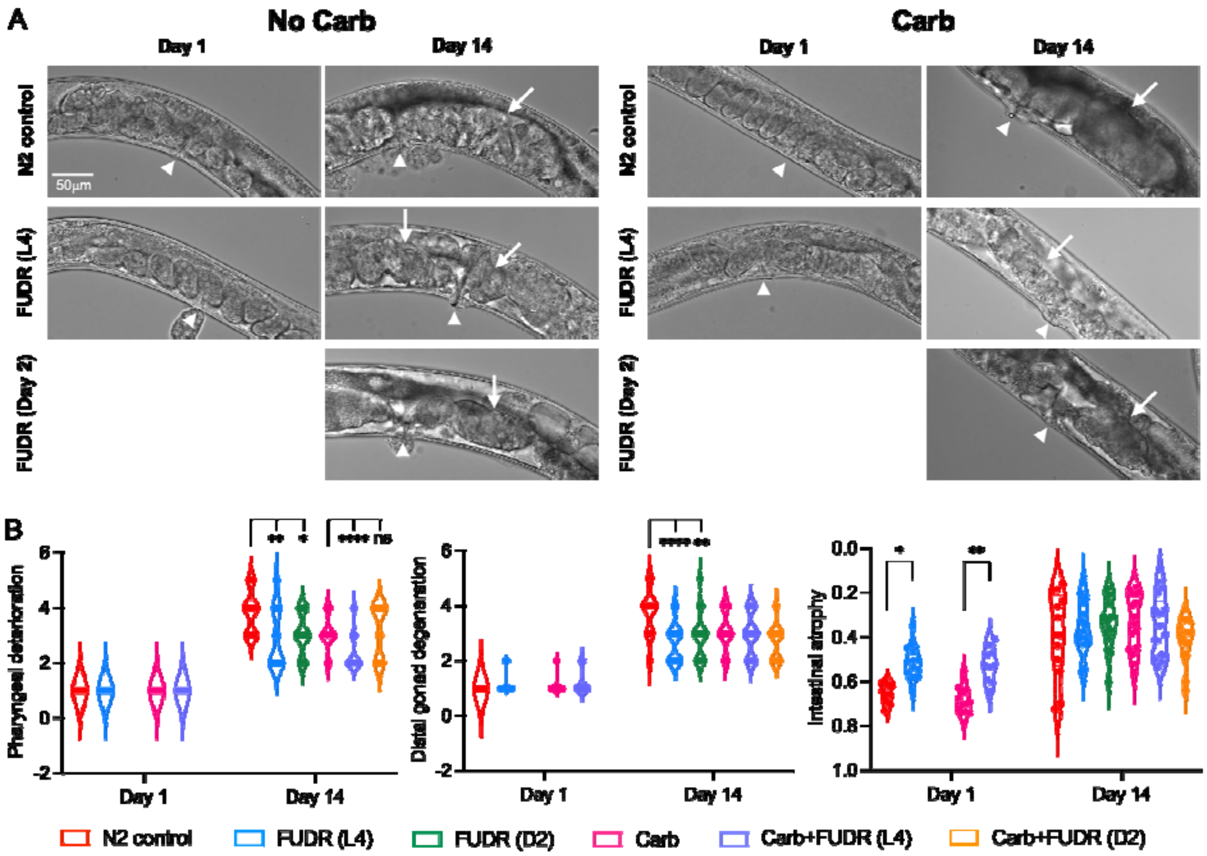

Supplementary Figure 1. Effects of FUDR on senescent pathologies in *C. elegans* hermaphrodites (20˚C). (**A**) Representative images of young and old nematodes. Arrows, uterine tumors; arrowheads, vulvae. (**B**) Effect of FUDR on three senescent pathologies. The apparent increase in intestinal atrophy present on D1 with FUDR from L4 likely reflects inhibition of intestinal growth.

bioRxiv preprint doi: https://doi.org/10.1101/2025.08.21.671442; this version posted August 25, 2025. The copyright holder for this preprint (which was not certified by peer review) is the author/funder, who has granted bioRxiv a license to display the preprint in perpetuity. It is made available under aCC-BY 4.0 International license.

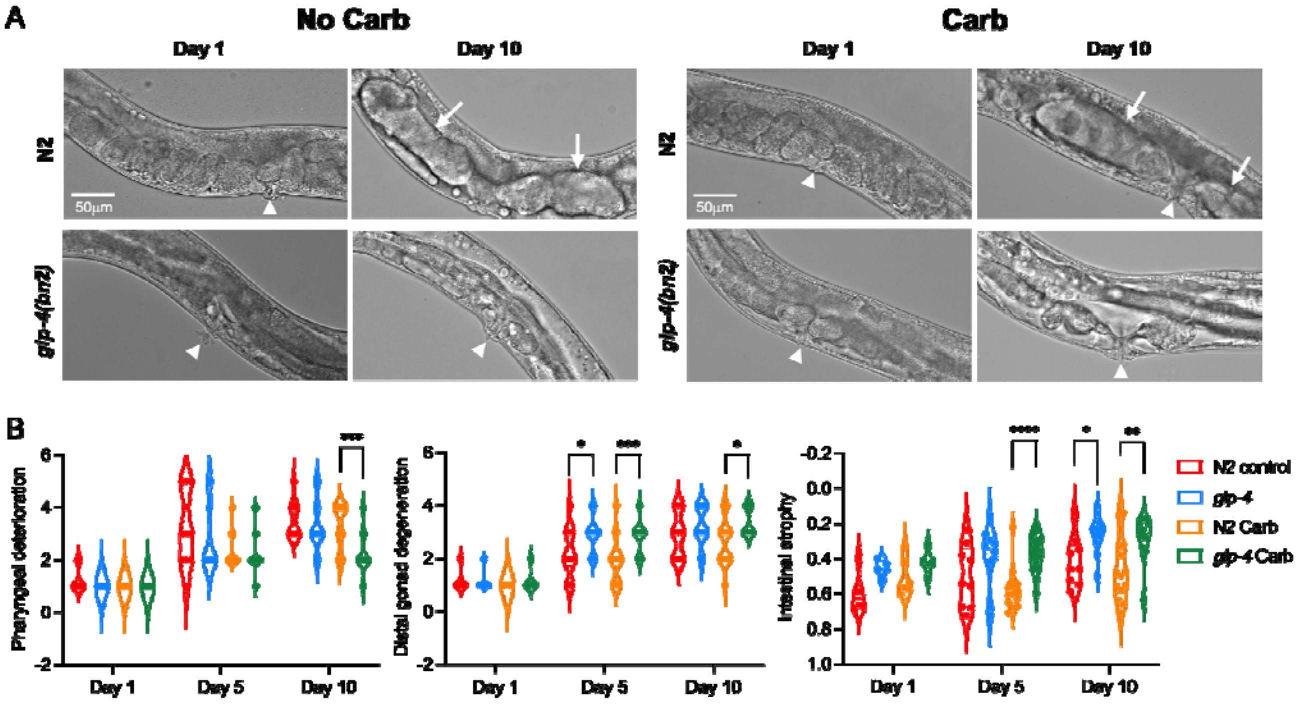

Supplementary Figure 2. Effects of *glp-4(bn2)* on aging pathologies (25˚C from L4). (**A**) Representative images of young and old nematodes. Arrows, uterine tumors (absent in old *glp-4* animal); arrowheads, vulvae. (**B**) Effects of *glp-4(bn2)* and Carb on three senescent pathologies.

bioRxiv preprint doi: https://doi.org/10.1101/2025.08.21.671442; this version posted August 25, 2025. The copyright holder for this preprint (which was not certified by peer review) is the author/funder, who has granted bioRxiv a license to display the preprint in perpetuity. It is made available under aCC-BY 4.0 International license.

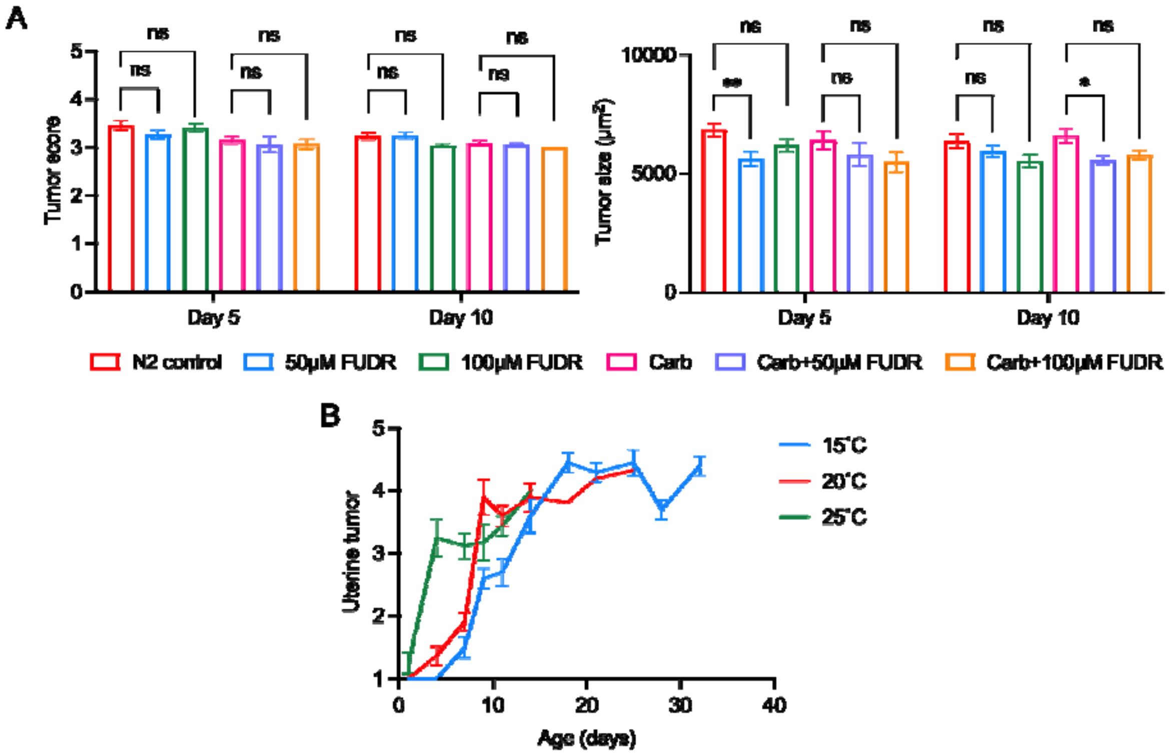

Supplementary Figure 3. Effects of FUDR on lifespan and uterine tumor development (25˚C). (**A**) FUDR does not increase lifespan in tumor-less *glp-4* mutant hermaphrodites. (**B**) Development of uterine tumor under different temperatures (15˚C, 20˚C, 25˚C). (C) At 25˚C, 50µM, 100µM FUDR only marginally reduce tumor size; tumor scale (left), tumor size (right). Measurement of tumor size (cross sectional area) is more accurate than the uterine tumor score.

bioRxiv preprint doi: https://doi.org/10.1101/2025.08.21.671442; this version posted August 25, 2025. The copyright holder for this preprint (which was not certified by peer review) is the author/funder, who has granted bioRxiv a license to display the preprint in perpetuity. It is made available under aCC-BY 4.0 International license.

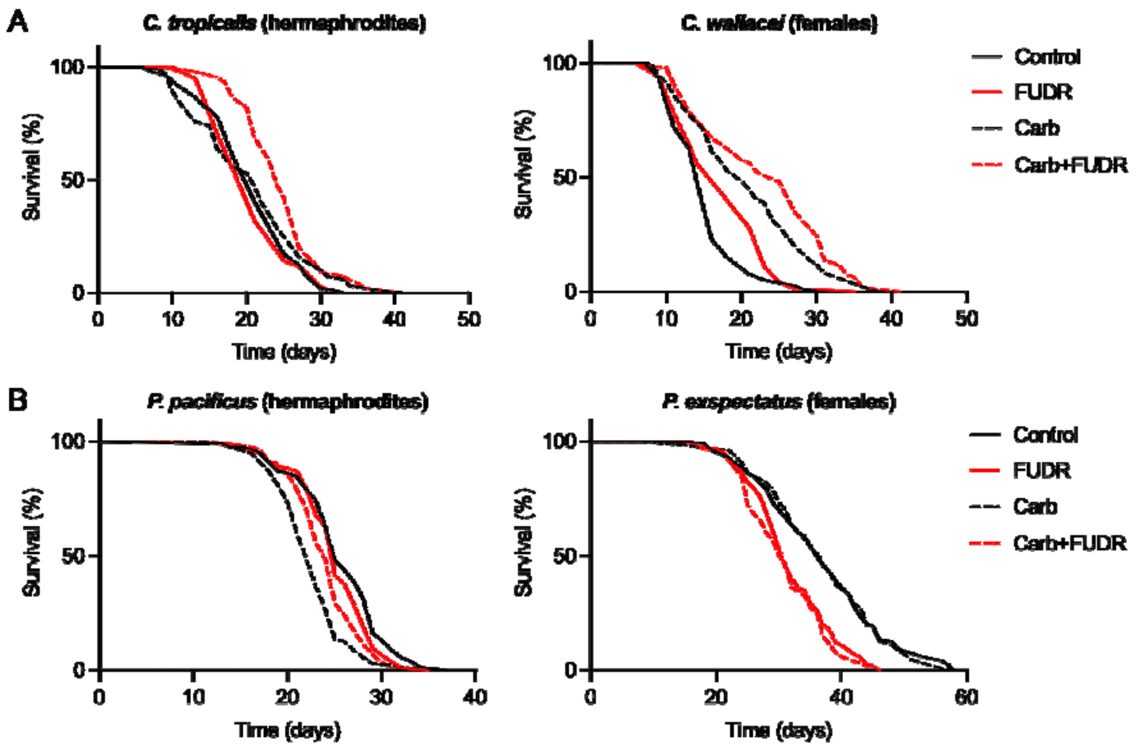

Supplementary Figure 4. Effects of FUDR on hermaphrodites and females of two sibling species pairs. (**A**) *Caenorhabditis tropicalis* (hermaphrodites) and *C. wallacei* (females). (**B**) *Pristionchus pacificus* (hermaphrodites) and *P. exspectatus* (females).

bioRxiv preprint doi: https://doi.org/10.1101/2025.08.21.671442; this version posted August 25, 2025. The copyright holder for this preprint (which was not certified by peer review) is the author/funder, who has granted bioRxiv a license to display the preprint in perpetuity. It is made available under aCC-BY 4.0 International license.

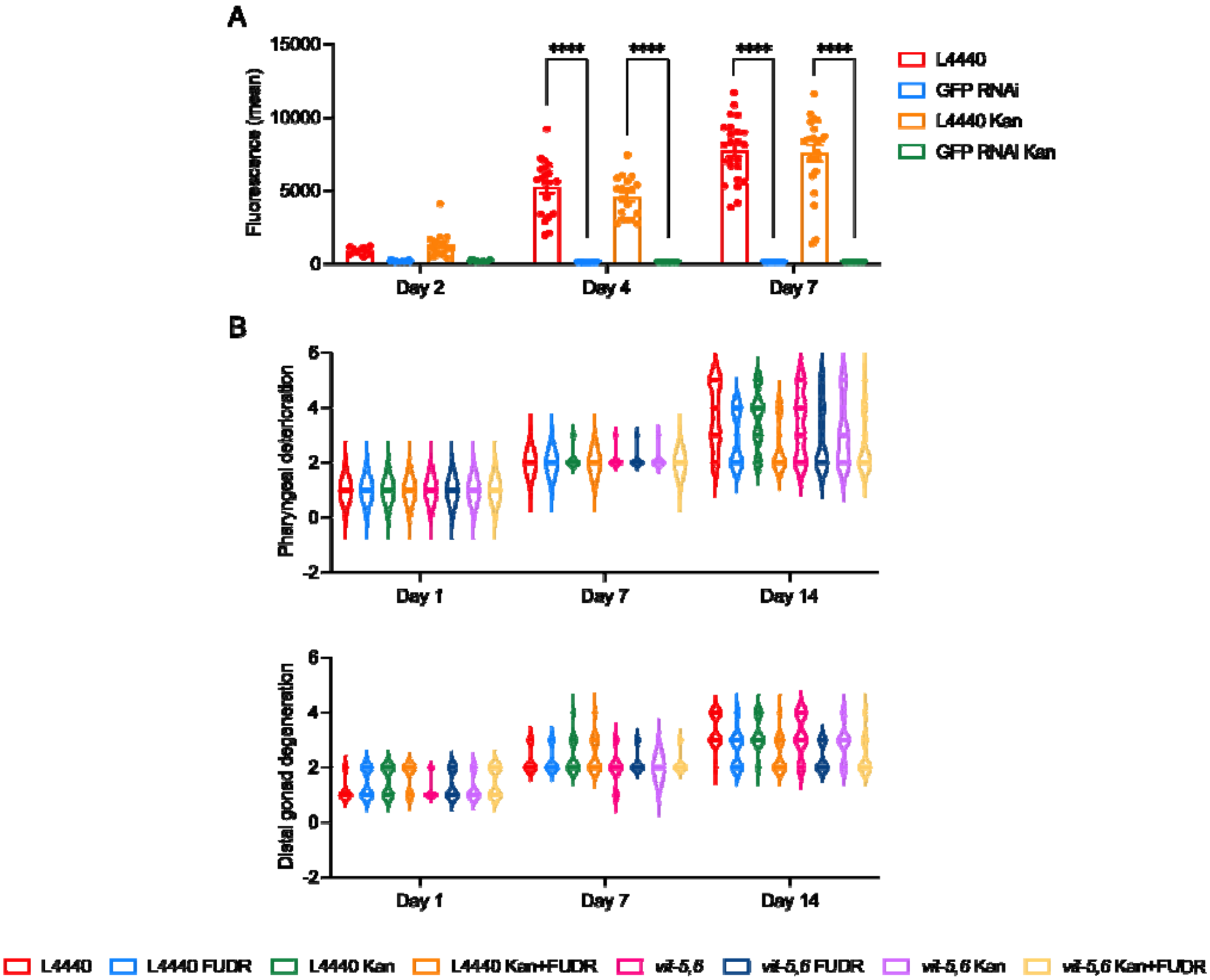

Supplementary Figure 5. Effects of treatment combinations to remove life-limiting pathologies. (**A**) No effect of Kan on knockdown of *pftn-1::gfp* by *gfp* RNAi. Error bars: standard error. (**B**) Effects of treatment combinations on pharyngeal deterioration and distal gonad degeneration.

bioRxiv preprint doi: https://doi.org/10.1101/2025.08.21.671442; this version posted August 25, 2025. The copyright holder for this preprint (which was not certified by peer review) is the author/funder, who has granted bioRxiv a license to display the preprint in perpetuity. It is made available under aCC-BY 4.0 International license.

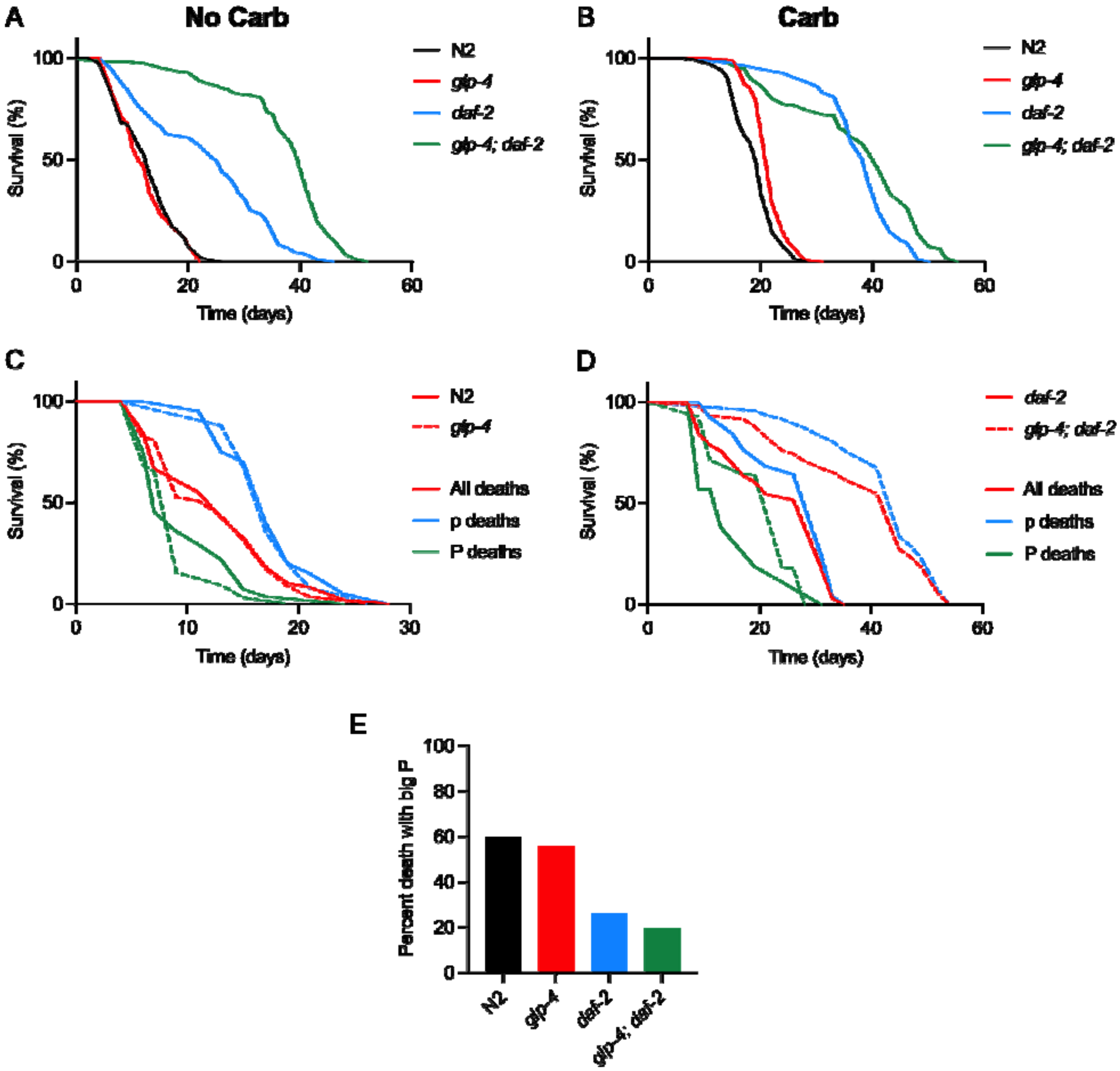

Supplementary Figure 6. *glp-4(bn2)* increases *daf-2(m577)* lifespan by increasing infection resistance (25˚C from L4 stage). (**A**, **B**) *glp-4* increases *daf-2(m577)* longevity in the absence (**A**) but not presence (**B**) of Carb. (**C**, **D**) Mortality deconvolution analysis of effects of *glp-4* and *daf-2*. Effects of *glp-4* in a wild-type (**C**) and *daf-2(m577)* (**D**) background (no Carb). (**E**) P death frequency is strongly reduced by *daf-2* but not *glp-4*.

bioRxiv preprint doi: https://doi.org/10.1101/2025.08.21.671442; this version posted August 25, 2025. The copyright holder for this preprint (which was not certified by peer review) is the author/funder, who has granted bioRxiv a license to display the preprint in perpetuity. It is made available under aCC-BY 4.0 International license.

Table S1. Effects of 50 mM FUDR on lifespan in the presence of Carb or Kan (20˚C)

Strain/ conditions	Number of deaths/ censored	Mean [median] lifespan (days)	% change vs. N2 control	p vs. N2 (log rank)	% change vs. non FUDR	p vs. non FUDR (log rank)
N2 control	**[C] 153/27**	**16.82 [16]**				
	[1] 54/8	17.31 [15]				
	[2] 51/9	17.29 [16]				
	[3] 50/10	15.82 [17]				
N2 FUDR (from L4)	**[C] 167/13**	**15.79 [15]**	**-6.12 [-6.25]**	**0.0024**	**-6.12 [-6.25]**	**0.0024**
	[1] 56/4	16.20 [15]	-6.41 [0]	0.0945	-6.41 [0]	0.0945
	[2] 57/3	15.88 [16]	-8.16 [0]	0.0397	-8.16 [0]	0.0397
	[3] 54/6	15.28 [14]	-3.41 [-17.65]	0.1805	-3.41 [-17.65]	0.1805
N2 FUDR (from D2)	**[C] 161/19**	**17.84 [19]**	**+6.06 [+18.75]**	**0.2742**	**+6.06 [+18.75]**	**0.2742**
	[1] 51/9	18.88 [21]	+9.07 [+40.00]	0.2787	+9.07 [+40.00]	0.2787
	[2] 55/5	17.65 [18]	+2.08 [+12.50]	0.9850	+2.08 [+12.50]	0.9850
	[3] 55/5	17.07 [17]	+7.90 [0]	0.3516	+7.90 [0]	0.3516
N2 Carb	**[C] 148/22**	**25.61 [25]**	**+52.26 [+56.25]**	**<0.0001**		
	[1] 40/10	23.50 [21]	+35.76 [+40.00]	<0.0001		
	[2] 52/8	24.54 [25]	+41.93 [+56.25]	<0.0001		
	[3] 56/4	28.11 [28]	+77.69 [+64.71]	<0.0001		
N2 Carb + FUDR (from L4)	**[C] 166/14**	**24.12 [24]**	**+43.40 [+50.00]**	**<0.0001**	**-5.82 [-4.00]**	**<0.0001**
	[1] 53/7	24.70 [25]	+42.69 [+66.67]	<0.0001	+5.11 [+19.05]	0.9100
	[2] 55/5	23.20 [23]	+34.18 [+43.75]	<0.0001	-5.46 [-8.00]	0.0267
	[3] 58/2	24.47 [24]	+54.68 [+41.18]	<0.0001	-12.95 [-14.29]	<0.0001
N2 Carb + FUDR (from D2)	**[C] 163/17**	**31.25 [32]**	**+85.79 [+100.00]**	**<0.0001**	**+22.02 [+28.00]**	**<0.0001**
	[1] 52/8	26.92 [27]	+55.52 [+80.00]	<0.0001	+14.55 [+28.57]	0.0359
	[2] 56/4	33.75 [35]	+95.20 [+118.75]	<0.0001	+37.53 [+40.00]	<0.0001
	[3] 55/5	32.80 [33]	+107.33 [+94.12]	<0.0001	+16.68 [+17.86]	0.0035
N2 Kan	**[C] 156/24**	**25.13 [25]**	**+49.41 [+56.25]**	**<0.0001**		
	[1] 46/14	22.87 [23]	+32.12 [+53.33]	<0.0001		
	[2] 56/4	27.32 [25]	+58.01 [+56.25]	<0.0001		
	[3] 54/6	24.80 [24]	+56.76 [+41.18]	<0.0001		
N2 Kan + FUDR (from L4)	**[C] 161/19**	**23.75 [24]**	**+41.20 [+50.00]**	**<0.0001**	**-5.49 [-4.00]**	**<0.0001**
	[1] 51/9	24.76 [25]	+43.04 [+66.67]	<0.0001	+8.26 [+8.70]	0.7003
	[2] 52/8	23.83 [25]	+37.83 [+56.25]	<0.0001	-12.77 [0]	0.0011
	[3] 58/2	22.78 [24]	+43.99 [+41.18]	<0.0001	-8.15 [0]	0.0085
N2 Kan + FUDR (from D2)	**[C] 141/39**	**29.61 [30]**	**+76.04 [+87.50]**	**<0.0001**	**+17.83 [+20.00]**	**<0.0001**
	[1] 44/16	28.84 [30]	+66.61 [+100.00]	<0.0001	+26.10 [+30.43]	<0.0001
	[2] 52/8	29.63 [30]	+71.37 [+87.50]	<0.0001	+8.46 [+20.00]	0.0779
	[3] 45/15	30.33 [31]	+91.72 [+82.35]	<0.0001	+22.30 [+29.17]	0.0019

bioRxiv preprint doi: https://doi.org/10.1101/2025.08.21.671442; this version posted August 25, 2025. The copyright holder for this preprint (which was not certified by peer review) is the author/funder, who has granted bioRxiv a license to display the preprint in perpetuity. It is made available under aCC-BY 4.0 International license.

Table S2. Effects of *glp-4(bn2)* on lifespan in the presence of Carb or Kan (25˚C from L4 stage)

**Strain/ condition**	**Number of deaths/ censored**	**Mean [median] lifespan (days)**	**% change vs. N2 control**	**p vs. N2 (log rank)**	**% change vs. non *glp-4***	**p vs. non *glp-4* (log rank)**
**Carbenicillin**						
N2 Control	**[C] 154/26**<br>[1] 49/11<br>[2] 54/6<br>[3] 51/9	**13.10 [14]**<br>12.69 [12]<br>13.39 [13]<br>13.16 [14]				
glp-4(bn2)	**[C] 169/11**<br>[1] 56/4<br>[2] 59/1<br>[3] 54/6	**11.45 [10]**<br>9.84 [8]<br>12.63 [12]<br>11.79 [12]	**-12.60 [-28.57]**<br>-22.46 [-33.33]<br>-5.68 [-7.69]<br>-10.41 [-14.29]	**0.0005**<br>0.0220<br>0.1984<br>0.0250	**-12.60 [-28.57]**<br>-22.46 [-33.33]<br>-5.68 [-7.69]<br>-10.41 [-14.29]	**0.0005**<br>0.0220<br>0.1984<br>0.0250
N2 Carb	**[C] 145/35**<br>[1] 48/12<br>[2] 51/9<br>[3] 46/14	**19.69 [21]**<br>20.04 [20]<br>20.40 [21]<br>18.50 [19]	**+50.31 [+50.00]**<br>+57.08 [+66.67]<br>+52.35 [+64.54]<br>+40.58 [+35.71]	**<0.0001**<br><0.0001<br><0.0001<br><0.0001		
glp-4(bn2) Carb	**[C] 163/17**<br>[1] 50/10<br>[2] 56/4<br>[3] 57/3	**21.19 [21]**<br>22.33 [24]<br>21.25 [21]<br>20.12 [21]	**+61.76 [+50.00]**<br>+75.97 [+100.00]<br>+58.70 [+61.54]<br>+52.89 [+50.00]	**<0.0001**<br><0.0001<br><0.0001<br><0.0001	**+7.62 [0]**<br>+11.43 [+20.00]<br>+4.17 [0]<br>+8.76 [+10.53]	**0.0012**<br>0.0034<br>0.2545<br>0.0456
**Kanamycin**						
N2 Control	**[C] 155/25**<br>[1] 47/13<br>[2] 52/8<br>[3] 56/4	**11.77 [10]**<br>10.57 [8]<br>12.10 [10]<br>12.51 [13]				
glp-4(bn2)	**[C] 171/9**<br>[1] 55/5<br>[2] 59/1<br>[3] 57/3	**11.52 [10]**<br>10.32 [8]<br>11.71 [10]<br>12.51 [13]	**-2.12 [0]**<br>-2.37 [0]<br>-3.22 [0]<br>0 [0]	**0.3174**<br>0.8202<br>0.4321<br>0.5910	**-2.12 [0]**<br>-2.37 [0]<br>-3.22 [0]<br>0 [0]	**0.3174**<br>0.8202<br>0.4321<br>0.5910
N2 Kan	**[C] 170/10**<br>[1] 56/4<br>[2] 55/5<br>[3] 59/1	**16.50 [18]**<br>16.00 [15]<br>18.02 [19]<br>15.56 [18]	**+40.19 [+80.00]**<br>+51.37 [+87.50]<br>+48.93 [+90. 00]<br>+24.38 [+38.46]	**<0.0001**<br><0.0001<br><0.0001<br>0.0022		
glp-4(bn2) Kan	**[C] 175/5**<br>[1] 58/2<br>[2] 57/3<br>[3] 60/0	**18.46 [19]**<br>19.16 [19]<br>20.00 [21]<br>16.33 [18]	**+56.84 [+90.00]**<br>+81.27 [+137.50]<br>+65.29 [+110.00]<br>+30.54 [+38.46]	**<0.0001**<br><0.0001<br><0.0001<br>0.0005	**+11.88 [+5.56]**<br>+19.75 [+26.67]<br>+10.99 [+10.53]<br>+4.95 [0]	**0.0221**<br>0.0062<br>0.1356<br>0.8742

bioRxiv preprint doi: https://doi.org/10.1101/2025.08.21.671442; this version posted August 25, 2025. The copyright holder for this preprint (which was not certified by peer review) is the author/funder, who has granted bioRxiv a license to display the preprint in perpetuity. It is made available under aCC-BY 4.0 International license.

Table S3. Effects of *daf-16* on *glp-4* longevity (25˚C from L4)

Strain/ conditions	Number of deaths/ censored	Mean [median] lifespan (days)	% change vs. control	p vs. N2 (log rank)	% change vs. non *glp-4*	p vs. non *glp-4* (log rank)
			**No carbenicillin**			
N2 control	**[C] 199/11**<br>[1] 52/8<br>[2] 29/1<br>[3] 60/0<br>[4] 58/2	**13.59 [14]**<br>15.37 [18]<br>14.66 [14]<br>10.92 [8]<br>14.24 [15]				
glp-4(bn2)	**[C] 265/5**<br>[1] 59/1<br>[2] 59/1<br>[3] 88/2<br>[4] 59/1	**11.57 [11]**<br>12.05 [11]<br>12.29 [13]<br>11.10 [10]<br>11.07 [11]	**-14.86 [-21.43]**<br>-21.60 [-38.89]<br>-16.17 [-7.14]<br>+1.65 [+25.00]<br>-22.26 [-26.67]	**<0.0001**<br><0.0001<br>0.0295<br>0.9090<br>0.0002	**-14.86 [-21.43]**<br>-21.60 [-38.89]<br>-16.17 [-7.14]<br>+1.65 [+25.00]<br>-22.26 [-26.67]	**<0.0001**<br><0.0001<br>0.0295<br>0.9090<br>0.0002
daf-16(mu86)	**[C] 171/9**<br><br>[2] 57/3<br>[3] 59/1<br>[4] 55/5	**9.30 [9]**<br><br>9.61 [9]<br>9.02 [8]<br>9.29 [8]	**-31.57 [-35.71]**<br><br>-34.45 [-35.71]<br>-17.40 [0]<br>-34.76 [-46.67]	**<0.0001**<br><br><0.0001<br>0.0098<br><0.0001		
daf-16(mu86) glp-4(bn2)	**[C] 206/4**<br>[1] 28/2<br>[2] 60/0<br>[3] 59/1<br>[4] 59/1	**9.60 [10]**<br>9.36 [9]<br>9.65 [9]<br>9.58 [10]<br>9.68 [11]	**-29.36 [-28.57]**<br>-39.10 [-50.00]<br>-34.17 [-35.71]<br>-12.27 [-25.00]<br>-32.02 [-26.67]	**<0.0001**<br><0.0001<br><0.0001<br>0.0615<br><0.0001	**+3.23 [+11.11]**<br><br>+0.42 [0]<br>+6.21 [+25.00]<br>+4.20 [+37.50]	**0.7531**<br><br>0.8486<br>0.4038<br>0.6256
			**Carbenicillin**			
N2 control	**[C] 223/12**<br>[1] 52/3<br>[2] 56/4<br>[3] 59/1<br>[4] 56/4	**21.52 [21]**<br>20.96 [21]<br>22.25 [24]<br>21.85 [22]<br>20.96 [21]				
glp-4(bn2)	**[C] 232/8**<br>[1] 59/1<br>[2] 58/2<br>[3] 59/1<br>[4] 56/4	**23.38 [24]**<br>22.69 [23]<br>24.28 [26]<br>24.02 [25]<br>22.48 [22]	**+8.46 [+14.29]**<br>+8.25 [+9.52]<br>+9.12 [+8.33]<br>+9.93 [+13.64]<br>+7.25 [+4.76]	**0.0001**<br>0.1711<br>0.0110<br>0.0096<br>0.1246	**+8.46 [+14.29]**<br>+8.25 [+9.52]<br>+9.12 [+8.33]<br>+9.93 [+13.64]<br>+7.25 [+4.76]	**0.0001**<br>0.1711<br>0.0110<br>0.0096<br>0.1246
daf-16(mu86)	**[C] 235/5**<br>[1] 59/1<br>[2] 60/0<br>[3] 58/2<br>[4] 58/2	**13.28 [13]**<br>13.88 [14]<br>13.25 [13]<br>13.16 [12]<br>12.81 [13]	**-38.30 [-38.10]**<br>-33.78 [-33.33]<br>-40.45 [-45.83]<br>-39.77 [-45.45]<br>-38.88 [-38.10]	**<0.0001**<br><0.0001<br><0.0001<br><0.0001<br><0.0001		
daf-16(mu86) glp-4(bn2)	**[C] 236/4**<br>[1] 58/2<br>[2] 59/1<br>[3] 60/0<br>[4] 59/1	**16.14 [16]**<br>15.76 [16]<br>16.02 [16]<br>18.28 [18]<br>14.46 [15]	**-25.00 [-23.81]**<br>-24.81 [-23.81]<br>-28.00 [-33.33]<br>-16.34 [-18.18]<br>-31.01 [-28.57]	**<0.0001**<br><0.0001<br><0.0001<br><0.0001<br><0.0001	**+21.54 [+23.08]**<br>+13.54 [+14.29]<br>+20.91 [+23.08]<br>+38.91 [+50.00]<br>+12.88 [+15.38]	**<0.0001**<br><0.0001<br><0.0001<br><0.0001<br><0.0001

bioRxiv preprint doi: https://doi.org/10.1101/2025.08.21.671442; this version posted August 25, 2025. The copyright holder for this preprint (which was not certified by peer review) is the author/funder, who has granted bioRxiv a license to display the preprint in perpetuity. It is made available under aCC-BY 4.0 International license.

**Strain/ conditions**	**Number of deaths/ censored**	**Mean [median] lifespan (days)**	**% change vs. control**	**p vs. N2 (log rank)**	**% change vs. non *glp-4***	**p vs. non *glp-4* (log rank)**
**No carbenicillin**						
N2 control	**[C] 244/26**<br>[1] 51/9<br>[2] 27/3<br>[3] 57/3<br>[4] 56/4<br>[5] 53/7	**13.13 [13]**<br>13.14 [12]<br>10.50 [11]<br>13.35 [13]<br>13.52 [14]<br>13.87 [13]				
glp-4(bn2)	**[C] 291/9**<br>[1] 59/1<br>[2] 59/1<br>[3] 59/1<br>[4] 55/5<br>[5] 59/1	**11.53 [11]**<br>11.08 [10]<br>11.69 [11]<br>11.37 [11]<br>11.69 [11]<br>11.81 [11]	**-12.19 [-15.38]**<br>-15.68 [-16.67]<br>+11.33 [0]<br>-14.83 [-15.38]<br>-13.54 [-21.43]<br>-14.85 [-15.38]	**<0.0001**<br>0.0203<br>0.5979<br>0.0139<br>0.1037<br>0.0177	**-12.19 [-15.38]**<br>-15.68 [-16.67]<br>+11.33 [0]<br>-14.83 [-15.38]<br>-13.54 [-21.43]<br>-14.85 [-15.38]	**<0.0001**<br>0.0203<br>0.5979<br>0.0139<br>0.1037<br>0.0177
daf-12(m20)	**[C] 211/29**<br>[1] 52/8<br>[2] 56/4<br><br>[4] 54/6<br>[5] 49/11	**10.02 [9]**<br>10.75 [9]<br>9.45 [8]<br><br>9.48 [8]<br>10.49 [9]	**-23.69 [-30.77]**<br>-18.19 [-25.00]<br>-10.00 [-27.27]<br><br>-29.88 [-42.86]<br>-24.37 [-30.77]	**<0.0001**<br>0.0111<br>0.0841<br><br><0.0001<br>0.0002		
daf-12(m20); glp-4(bn2)	**[C] 283/17**<br>[1] 59/1<br>[2] 60/0<br>[3] 52/8<br>[4] 59/1<br>[5] 53/7	**10.00 [10]**<br>10.47 [10]<br>9.85 [11]<br>10.19 [11]<br>9.92 [11]<br>9.49 [9]	**-23.84 [-23.08]**<br>-20.32 [-16.67]<br>-6.19 [0]<br>-23.67 [-15.38]<br>-26.63 [-21.43]<br>-31.58 [-30.77]	**<0.0001**<br>0.0017<br>0.0481<br>0.0001<br><0.0001<br><0.0001	**-0.20 [+11.11]**<br>-2.60 [+11.11]<br>+4.23 [+37.50]<br><br>+4.64 [+37.50]<br>-9.53 [0]	**0.2175**<br>0.6592<br>0.9437<br><br>0.7131<br>0.0430
**Carbenicillin**						
N2 control	**[C] 273/27**<br>[1] 58/2<br>[2] 56/4<br>[3] 59/1<br>[4] 53/7<br>[5] 47/13	**20.85 [21]**<br>22.72 [23]<br>18.38 [19]<br>20.75 [21]<br>20.72 [21]<br>21.79 [22]				
glp-4(bn2)	**[C] 281/19**<br>[1] 60/0<br>[2] 58/2<br>[3] 52/8<br>[4] 60/0<br>[5] 51/9	**22.76 [23]**<br>24.00 [23]<br>19.53 [19]<br>24.13 [25]<br>22.85 [23]<br>23.49 [24]	**+9.16 [+9.52]**<br>+5.63 [0]<br>+6.26 [0]<br>+16.29 [+19.04]<br>+10.28 [+9.52]<br>+7.80 [+9.09]	**<0.0001**<br>0.0271<br>0.0995<br><0.0001<br>0.0068<br>0.2444	**+9.16 [+9.52]**<br>+5.63 [0]<br>+6.26 [0]<br>+16.29 [+19.04]<br>+10.28 [+9.52]<br>+7.80 [+9.09]	**<0.0001**<br>0.0271<br>0.0995<br><0.0001<br>0.0068<br>0.2444
daf-12(m20)	**[C] 276/24**<br>[1] 55/5<br>[2] 57/3<br>[3] 56/4<br>[4] 56/4<br>[5] 52/8	**15.87 [16]**<br>17.49 [18]<br>14.77 [14]<br>16.16 [16]<br>14.84 [14]<br>16.15 [16]	**-23.88 [-23.81]**<br>-23.02 [-21.74]<br>-19.64 [-26.32]<br>-22.12 [-23.81]<br>-28.39 [-33.33]<br>-25.88 [-27.27]	**<0.0001**<br><0.0001<br><0.0001<br><0.0001<br><0.0001<br><0.0001		
daf-12(m20); glp-4(bn2)	**[C] 276/24**<br>[1] 40/20<br>[2] 60/0<br>[3] 60/0<br>[4] 60/0<br>[5] 56/4	**17.57 [18]**<br>18.88 [18]<br>15.40 [14]<br>17.85 [18]<br>17.38 [16]<br>18.88 [19]	**-15.73 [-14.29]**<br>-16.90 [-21.74]<br>-16.21 [-26.32]<br>-13.98 [-14.29]<br>-16.12 [-23.81]<br>-13.35 [-13.64]	**<0.0001**<br>0.0017<br><0.0001<br><0.0001<br><0.0001<br><0.0001	**+10.71 [+12.50]**<br>+7.95 [0]<br>+4.27 [0]<br>+10.46 [+12.50]<br>+17.12 [+14.29]<br>+16.90 [+18.75]	**<0.0001**<br><0.0001<br>0.2835<br>0.0013<br>0.0002<br><0.0001

Table S4. Effects of *daf-12* on *glp-4* longevity (25˚C from L4)

bioRxiv preprint doi: https://doi.org/10.1101/2025.08.21.671442; this version posted August 25, 2025. The copyright holder for this preprint (which was not certified by peer review) is the author/funder, who has granted bioRxiv a license to display the preprint in perpetuity. It is made available under aCC-BY 4.0 International license.

Table S5. Effects of FUDR on *glp-4(bn2)* lifespan (25˚C from L4 stage)

Strain/ condition	Number of deaths/ censored	Mean [median] lifespan (days)	% change vs. N2 control	p vs. N2 control (log rank)	% change vs. non *glp-4*	p vs. non *glp-4* (log rank)	% change vs. untreated	p vs. untreated (log rank)
**No carbenicillin**								
N2 control	**[C] 114/16**<br>[1] 26/4<br>[2] 40/0<br>[3] 48/12	**12.14 [11]**<br>11.87 [11]<br>11.95 [11]<br>12.38 [12]						
N2 FUDR (D2)	**[C] 139/21**<br>[1] 52/8<br>[2] 40/0<br>[3] 47/13	**11.70 [12]**<br>11.74 [13]<br>11.68 [12]<br>11.71 [12]	**-3.62 [+9.09]**<br>-1.10 [+18.18]<br>-2.26 [9.09]<br>-5.41 [0]	**0.1672**<br>0.6440<br>0.4558<br>0.4450			**-3.62 [+9.09]**<br>-1.10 [+18.18]<br>-2.26 [9.09]<br>-5.41 [0]	**0.1672**<br>0.6440<br>0.4558<br>0.4450
glp-4(bn2)	**[C] 177/3**<br>[1] 60/0<br>[2] 60/0<br>[3] 57/3	**10.78 [10]**<br>10.62 [8]<br>10.55 [8]<br>11.19 [10]	**-11.20 [-9.09]**<br>-10.53 [-27.27]<br>-11.72 [-27.27]<br>-9.61 [-16.67]	**0.0129**<br>0.2372<br>0.1610<br>0.1061	**-11.20 [-9.09]**<br>-10.53 [-27.27]<br>-11.72 [-27.27]<br>-9.61 [-16.67]	**0.0129**<br>0.2372<br>0.1610<br>0.1061		
glp-4(bn2) FUDR (D2)	**[C] 179/1**<br>[1] 59/1<br>[2] 60/0<br>[3] 60/0	**10.48 [10]**<br>9.86 [8]<br>11.05 [11]<br>10.52 [10]	**-13.67 [-9.09]**<br>-16.93 [-27.27]<br>-7.53 [0]<br>-15.02 [-16.67]	**0.0006**<br>0.0473<br>0.1839<br>0.0143	**-10.43 [-16.67]**<br>-16.01 [-38.46]<br>-5.39 [-8.33]<br>-10.16 [-16.67]	**0.0169**<br>0.0218<br>0.5738<br>0.0847	**-2.78 [0]**<br>-7.16 [0]<br>+4.74 [+37.50]<br>-5.99 [0]	**0.3262**<br>0.2510<br>0.8902<br>0.3383
**Carbenicillin**								
N2 Control	**[C] 143/17**<br>[1] 39/1<br>[2] 45/15<br>[3] 59/1	**19.15 [20]**<br>18.97 [18]<br>19.71 [20]<br>18.85 [20]						
N2 FUDR (D2)	**[C] 132/18**<br>[1] 37/3<br>[2] 44/6<br>[3] 51/9	**20.97 [20]**<br>21.17 [23]<br>20.43 [20]<br>21.27 [20]	**+9.50 [0]**<br>+11.60 [+27.78]<br>+3.65 [0]<br>+12.84 [0]	**0.0032**<br>0.0399<br>0.7487<br>0.0104			**+9.50 [0]**<br>+11.60 [+27.78]<br>+3.65 [0]<br>+12.84 [0]	**0.0032**<br>0.0399<br>0.7487<br>0.0104
glp-4(bn2)	**[C] 170/10**<br>[1] 58/2<br>[2] 59/1<br>[3] 53/7	**21.57 [20]**<br>21.03 [20]<br>22.03 [23]<br>21.66 [20]	**+12.64 [0]**<br>+10.86 [+11.11]<br>+11.77 [+15.00]<br>+14.91 [0]	**<0.0001**<br>0.0500<br>0.0240<br>0.0021	**+12.64 [0]**<br>+10.86 [+11.11]<br>+11.77 [+15.00]<br>+14.91 [0]	**<0.0001**<br>0.0500<br>0.0240<br>0.0021		
glp-4(bn2) FUDR (D2)	**[C] 178/2**<br>[1] 60/0<br>[2] 60/0	20.43 [20]<br>20.05 [20]<br>21.27 [23]	**+6.68 [0]**<br>+5.69 [+11.11]<br>+7.91 [+15.00]	**0.0615**<br>0.4077<br>0.2421	**-2.58 [0]**<br>-5.29 [-13.04]<br>+4.11 [+15.00]	**0.1203**<br>0.0601<br>0.3464	**-5.29 [0]**<br>-4.66 [0]<br>-3.45 [0]	**0.0031**<br>0.1408<br>0.1595

bioRxiv preprint doi: https://doi.org/10.1101/2025.08.21.671442; this version posted August 25, 2025. The copyright holder for this preprint (which was not certified by peer review) is the author/funder, who has granted bioRxiv a license to display the preprint in perpetuity. It is made available under aCC-BY 4.0 International license.

	[3] 58/2	19.97 [20]	+5.94 [0]	0.2855	-6.11 [0]	0.0929	-7.80 [0]	0.0242

bioRxiv preprint doi: https://doi.org/10.1101/2025.08.21.671442; this version posted August 25, 2025. The copyright holder for this preprint (which was not certified by peer review) is the author/funder, who has granted bioRxiv a license to display the preprint in perpetuity. It is made available under aCC-BY 4.0 International license.

bioRxiv preprint doi: https://doi.org/10.1101/2025.08.21.671442; this version posted August 25, 2025. The copyright holder for this preprint (which was not certified by peer review) is the author/funder, who has granted bioRxiv a license to display the preprint in perpetuity. It is made available under aCC-BY 4.0 International license.

Table S6. Verification of effects of FUDR on *glp-4(bn2)* lifespan (25˚C from L4 stage)

Strain/ conditions	Number of deaths/ censored	Mean [median] lifespan (days)	% change vs. N2 Carb	p vs. N2 Carb (log rank)
N2 Carb	**[C] 303/22**	**18.14 [18]**		
	[1] 39/1	18.97 [18]		
	[2] 45/15	19.71 [20]		
	[3] 59/1	18.85 [20]		
	[4] 59/1	20.58 [21]		
	[5] 58/2	15.36 [16]		
	[6] 43/2	15.19 [14]		
N2	**[C] 312/18**	**19.34 [19]**	**+6.62 [+5.56]**	**0.0065**
Carb + FUDR (D2)	[1] 37/3	21.17 [23]	+11.60 [+27.78]	0.0399
	[2] 44/6	20.43 [20]	+3.65 [0]	0.7487
	[3] 51/9	21.27 [20]	+12.84 [0]	0.0104
	[4] 60/0	21.08 [21]	+2.43 [0]	0.3715
	[5] 60/0	16.38 [16]	+6.64 [0]	0.1037
	[6] 60/0	17.02 [16]	+12.05 [+14.29]	0.0103

bioRxiv preprint doi: https://doi.org/10.1101/2025.08.21.671442; this version posted August 25, 2025. The copyright holder for this preprint (which was not certified by peer review) is the author/funder, who has granted bioRxiv a license to display the preprint in perpetuity. It is made available under aCC-BY 4.0 International license.

Table S7. Effects of 50 mM FUDR on lifespan of N2 hermaphrodites and males in monoxenic liquid culture (20˚C, monoxenic liquid culture).

Strain/ condition	Number of deaths/ censored	Mean [median] lifespan (days)	% change vs. N2 control	*p* vs. N2 control (log rank)	% change vs. untreated	*p* vs. untreated (log rank)
***C. elegans* (hermaphrodites)**						
N2 control	**[C] 13/131**<br>[1] 5/43<br>[2] 6/42<br>[3] 2/46	**15.95[1] [15]**<br>13.98 [11]<br>16.30 [15]<br>17.59 [15]				
N2 FUDR (D2)	**[C] 3/141**<br>[1] 3/45<br>[2] 0/48<br>[3] 0/48	**20.34 [20]**<br>18.56 [18]<br>20.77 [22]<br>21.56 [22]	**+27.52 [+33.33]**<br>+32.76 [+63.64]<br>+27.42 [+46.67]<br>+22.57 [+46.67]	**<0.0001**<br><0.0001<br>0.0010<br>0.0042	**+27.52 [+33.33]**<br>+32.76 [+63.64]<br>+27.42 [+46.67]<br>+22.57 [+46.67]	**<0.0001**<br><0.0001<br>0.0010<br>0.0042
N2 Carb	**[C] 22/122**<br>[1] 9/39<br>[2] 8/40<br>[3] 5/43	**23.91 [25]**<br>22.62 [23]<br>23.91 [26]<br>25.07 [25]	**+49.91 [+66.67]**<br>+61.80 [+109.09]<br>+46.69 [+73.33]<br>+42.52 [+66.67]	**<0.0001**<br><0.0001<br><0.0001<br><0.0001		
N2 Carb FUDR (D2)	**[C] 19/125**<br>[1] 7/41<br>[2] 1/47<br>[3] 11/37	**29.19 [31]**<br>27.93 [30]<br>29.82 [33]<br>29.91 [32]	**+83.01[+106.67]**<br>+99.79 [+172.72]<br>+82.94 [+120.00]<br>+70.04 [+113.33]	**<0.0001**<br><0.0001<br><0.0001<br><0.0001	**+22.08 [+24.00]**<br>+23.47 [+30.43]<br>+24.72 [+26.92]<br>+19.31 [+28.00]	**<0.0001**<br><0.0001<br><0.0001<br>0.0012
***C. elegans* (males)**						
N2 control	**[C] 5/139**<br>[1] 4/44<br>[2] 0/48<br>[3] 1/47	**13.48[1] [12]**<br>12.53 [13]<br>12.71 [12]<br>15.24 [11]				
N2 FUDR (D2)	**[C] 4/140**<br>[1] 1/47<br>[2] 2/46<br>[3] 1/47	**15.14 [15]**<br>12.04 [11]<br>16.84 [17]<br>16.56 [15]	**+12.31 [+25.00]**<br>-3.91 [-15.38]<br>+32.49 [+41.67]<br>+8.661[+36.36]	**0.0027**<br>0.5591<br><0.0001<br>0.8250	**+12.31 [+25.00]**<br>-3.91 [-15.38]<br>+32.49 [+41.67]<br>+8.66 [+36.36]	**0.0027**<br>0.5591<br><0.0001<br>0.8250
N2 Carb	**[C] 16/128**<br>[1] 5/43<br>[2] 11/37<br>[3] 0/48	**16.60 [18]**<br>15.11 [16]<br>15.35 [17]<br>19.29 [20]	**+23.15 [+50.00]**<br>+20.59 [+23.08]<br>+20.77 [+41.67]<br>+26.57 [+81.82]	**0.0001**<br>0.0591<br>0.0049<br>0.1031		
N2 Carb FUDR (D2)	**[C] 9/136**<br>[1] 6/42<br>[2] 1/47<br>[3] 2/46	**17.45 [18]**<br>14.50 [16]<br>17.13 [17]<br>20.53 [22]	**+29.45 [+50.00]**<br>+15.72 [+23.08]<br>+34.78 [+41.67]<br>+34.71 [+100.00]	**<0.0001**<br>0.2128<br>0.0001<br>0.0088	**+5.12 [0]**<br>-4.04 [0]<br>+11.60 [0]<br>+6.43 [+10]	**0.5883**<br>0.2413<br>0.1236<br>0.1362

[1]Note that in none of the three trials do solitary males live longer than solitary hermaphrodites. This is surprising given earlier studies that observed that solitary N2 males are longer-lived than hermaphrodites, either on NGM plates (Gems and Riddle, 2000) or in liquid culture (McCulloch and Gems, 2003; McCulloch and Gems, 2007). Moreover, greater male longevity is a feature of many free-living nematodes (McCulloch and Gems, 2003). We attribute the difference between earlier observations and those in the present study to an unknown difference in culture conditions. We note that the mean lifespans of the controls are somewhat lower than usual in these trials.

bioRxiv preprint doi: https://doi.org/10.1101/2025.08.21.671442; this version posted August 25, 2025. The copyright holder for this preprint (which was not certified by peer review) is the author/funder, who has granted bioRxiv a license to display the preprint in perpetuity. It is made available under aCC-BY 4.0 International license.

Table S8. Effects of FUDR on lifespan when administered only after tumor development.

Strain/ conditions	Number of deaths/ censored	Mean [median] lifespan (days)	% change vs. N2 Carb	p vs. N2 Carb (log rank)
N2 Carb	**[C] 85/5**<br>[1] 55/5<br>[2] 30/0	**24.04 [24]**<br>25.42 [24]<br>21.50 [22]		
N2<br>Carb + FUDR (D2)	**[C] 120/0**<br>[1] 60/0<br>[2] 60/0	**27.68 [28]**<br>29.05 [29.5]<br>26.30 [26.5]	**+15.14 [+16.67]**<br>+14.28 [+22.92]<br>+22.33 [+20.45]	**0.0003**<br>0.0053<br>0.0001
N2<br>Carb + FUDR (D12)	**[C] 108/12**<br>[1] 56/4<br>[2] 52/8	**25.59 [26]**<br>25.84 [26]<br>25.33 [25]	**+6.45 [+8.33]**<br>+1.65 [+8.33]<br>+17.81[+13.64]	**0.0284**<br>0.3655<br>0.0044
N2<br>Carb + FUDR (D18)	**[C] 110/10**<br>[1] 55/5<br>[2] 55/5	**24.39 [24]**<br>22.42 [21]<br>26.36 [25]	**+1.46 [0]**<br>-11.80 [-12.50]<br>+22.60 [+13.64]	**0.3078**<br>0.1266<br>0.0002

bioRxiv preprint doi: https://doi.org/10.1101/2025.08.21.671442; this version posted August 25, 2025. The copyright holder for this preprint (which was not certified by peer review) is the author/funder, who has granted bioRxiv a license to display the preprint in perpetuity. It is made available under aCC-BY 4.0 International license.

Table S9. Effects of FUDR on lifespan in hermaphrodites and females of different free-living nematode species (*Caenorhabditis* species).

Strain/ condition	Number of deaths/ censored	Mean [median] lifespan (days)	% change vs. control	p vs. N2 control (log rank)	% change vs. untreated	p vs. untreated (log rank)
***C. tropicalis* (hermaphrodites)**						
Control	**[C] 111/9**<br>[1] 57/3<br>[2] 54/6	**20.58 [21]**<br>21.14 [21]<br>19.38 [21]				
FUDR (D2)	**[C] 88/32**<br>[1] 39/21<br>[2] 49/11	**19.95 [21]**<br>18.67 [21]<br>20.98 [21]	**-3.06 [0]**<br>-11.68 [0]<br>+8.26 [0]	**0.5760**<br>0.0267<br>0.4915	**-3.06 [0]**<br>-11.68 [0]<br>+8.26 [0]	**0.5760**<br>0.0267<br>0.4915
Carb	**[C] 106/14**<br>[1] 55/5<br>[2] 51/9	**20.25 [21]**<br>21.87 [22]<br>18.49 [18]	**-1.60 [0]**<br>+3.45 [+4.76]<br>-4.59 [-14.29]	**0.4583**<br>0.0746<br>0.2912		
Carb FUDR (D2)	**[C] 98/22**<br>[1] 48/12<br>[2] 50/10	**24.46 [24]**<br>25.73 [27]<br>23.24 [23]	**+18.85 [+14.29]**<br>+21.71 [+28.57]<br>+19.92 [+9.52]	**<0.0001**<br>0.0001<br>0.0089	**+20.79 [+14.29]**<br>+17.65 [+22.73]<br>+25.69 [+27.78]	**0.0022**<br>0.0959<br>0.0004
***C. wallacei* (females)**						
Control	**[C] 81/39**<br>[1] 46/14<br>[2] 35/25	**15.04 [14]**<br>15.72 [15]<br>14.14 [13]				
FUDR (D2)	**[C] 75/15**<br>[1] 28/2<br>[2] 47/13	**17.13 [16]**<br>18.07 [18]<br>16.57 [16]	**+13.90 [+14.29]**<br>+14.95 [+20.00]<br>+17.19 [+23.08]	**0.0127**<br>0.0185<br>0.0731	**+13.90 [+14.29]**<br>+14.95 [+20.00]<br>+17.19 [+23.08]	**0.0127**<br>0.0185<br>0.0731
Carb	**[C] 89/31**<br>[1] 46/14<br>[2] 43/17	**20.28 [20]**<br>22.63 [24]<br>17.77 [16]	**+34.84 [+42.86]**<br>+43.96 [+60.00]<br>+25.67 [+23.08]	**<0.0001**<br><0.0001<br>0.0127		
Carb FUDR (D2)	**[C] 95/15**<br>[1] 55/5<br>[2] 40/10	22.98 [24]<br>25.29 [27]<br>19.80 [16]	**+52.79 [+71.43]**<br>+60.88 [+80.00]<br>+40.03 [+23.08]	**<0.0001**<br><0.0001<br>0.0023	**+13.31 [+20.00]**<br>+11.75 [+12.50]<br>+11.42 [0]	**0.0465**<br>0.1424<br>0.3423

bioRxiv preprint doi: https://doi.org/10.1101/2025.08.21.671442; this version posted August 25, 2025. The copyright holder for this preprint (which was not certified by peer review) is the author/funder, who has granted bioRxiv a license to display the preprint in perpetuity. It is made available under aCC-BY 4.0 International license.

Table S10. Effects of FUDR on lifespan in hermaphrodites and females of different free-living nematode species (*Pristionchus* species).

Strain/ condition	Number of deaths/ censored	Mean [median] lifespan (days)	% change vs. control	p vs. N2 control (log rank)	% change vs. untreated	p vs. untreated (log rank)
P. pacificus **(hermaphrodites)**						
control	**[C] 122/3**<br>[1] 30/0<br>[2] 34/1<br>[3] 58/2	**25.61 [25]**<br>26.03 [25]<br>25.06 [25]<br>25.72 [26]				
FUDR (D2)	**[C] 183/25**<br>[1] 59/1<br>[2] 64/1<br>[3] 60/0	**25.03 [25]**<br>24.34 [25]<br>25.55 [25]<br>25.15 [25]	**-2.26 [0]**<br>-6.49 [0]<br>+1.96 [0]<br>-2.22 [-3.85]	**0.0324**<br>0.0520<br>0.6329<br>0.4218	**-2.26 [0]**<br>-6.49 [0]<br>+1.96 [0]<br>-2.22 [-3.85]	**0.0324**<br>0.0520<br>0.6329<br>0.4218
Carb	**[C] 189/11**<br>[1] 62/8<br>[2] 67/3<br>[3] 60/0	**22.50 [22]**<br>22.77 [23]<br>21.66 [21]<br>23.17 [22]	**-12.14 [-12.00]**<br>-12.52 [-8.00]<br>-13.57 [-16.00]<br>-9.91 [-15.38]	**<0.0001**<br>0.0023<br><0.0001<br>0.0004		
Carb FUDR (D2)	**[C] 197/3**<br>[1] 68/2<br>[2] 69/1<br>[3] 60/0	**24.10 [24]**<br>23.65 [25]<br>24.10 [25]<br>24.62 [24]	**-5.90 [-4.00]**<br>-9.14 [0]<br>-3.83 [0]<br>-4.28 [-7.69]	**<0.0001**<br>0.0038<br>0.0286<br>0.0652	**+7.11 [+9.09]**<br>+3.86 [+8.70]<br>+11.27 [+19.05]<br>+6.26 [+9.09]	**0.0001**<br>0.2894<br><0.0001<br>0.0366
P. exspectatus **(females)**						
control	**[C] 121/24**<br>[1] 22/8<br>[2] 51/9<br>[3] 48/7	**36.59 [36]**<br>35.18 [37]<br>39.00 [39]<br>34.67 [36]				
FUDR (D2)	**[C] 155/15**<br>[1] 39/11<br>[2] 59/1<br>[3] 57/3	**31.69 [31]**<br>31.67 [30]<br>33.14 [32]<br>30.21 [31]	**-13.39 [-13.89]**<br>-9.98 [-18.92]<br>-15.03 [-17.95]<br>-12.86 [-13.89]	**<0.0001**<br>0.0179<br><0.0001<br>0.0011	**-13.39 [-13.89]**<br>-9.98 [-18.92]<br>-15.03 [-17.95]<br>-12.86 [-13.89]	**<0.0001**<br>0.0179<br><0.0001<br>0.0011
Carb	**[C] 127/28**<br>[1] 40/10<br>[2] 49/11<br>[3] 38/7	**36.52 [37]**<br>37.18 [37]<br>38.88 [39]<br>32.97 [33]	**-0.19 [+2.78]**<br>+5.69 [0]<br>-0.31 [0]<br>-4.90 [-8.33]	**0.7435**<br>0.4291<br>0.6199<br>0.4102		
Carb FUDR (D2)	**[C] 150/15**<br>[1] 50/10<br>[2] 58/2<br>[3] 42/3	**30.80 [31]**<br>30.32 [32]<br>31.38 [31]<br>30.57 [31]	**-15.82 [-13.89]**<br>-13.81 [-13.51]<br>-19.54 [-20.51]<br>-11.83 [-13.89]	**<0.0001**<br>0.0047<br><0.0001<br>0.0108	**-15.66 [-16.22]**<br>-18.45 [-13.51]<br>-19.29 [-20.51]<br>-7.28 [-6.06]	**<0.0001**<br><0.0001<br><0.0001<br>0.0765

Table S11. Effects of *vit-5,-6* RNAi, Kan and 50 μM FUDR on senescent pathologies (statistical comparisons).

Strain/ conditions	Time	Pharyngeal deterioration			Distal gonad degeneration			Uterine tumor			Intestinal atrophy			Yolk pool accumulation		
		p vs. non FUDR	*p* vs. L4440 treatment	*p* vs. non Kan	*p* vs. non FUDR	*p* vs. L4440 treatment	*p* vs. non Kan	*p* vs. non FUDR	*p* vs. L4440 treatment	p vs. non Kan	p vs. non FUDR	p vs. L4440 treatment	p vs. non Kan	p vs. non FUDR	p vs. L4440 treatment	p vs. non Kan
L4440	Day 1															
	Day 7															
	Day14															
vit-5,-6 RNAi	Day 1		>0.99			0.99			>0.99			0.81			>0.99	
	Day 7		0.99			0.32			**<0.0001**			**0.0022**			0.106	
	Day14		0.47			0.42			**<0.0001**			**0.0010**			**0.0023**	
L4440 FUDR	Day 1	>0.99			0.37			>0.99			**0.028**			0.99		
	Day 7	>0.99			>0.99			**<0.0001**			0.83			**0.033**		
	Day14	**0.0023**			**<0.0001**			0.72			0.35			**0.0069**		
vit-5,-6 RNAi FUDR	Day 1	>0.99	>0.99		0.81	0.97		>0.99	>0.99		**0.0089**	0.82		0.88	0.91	
	Day 7	>0.99	0.99		0.54	0.95		**<0.0001**	**0.032**		**0.0003**	0.83		*0.099*	0.59	
	Day14	0.44	0.93		**0.0002**	0.25		0.98	0.47		0.98	*0.068*		**0.0008**	**0.0028**	
L4440 Kan	Day 1			>0.99			0.23			>0.99			0.98			0.76
	Day 7			0.93			0.84			0.84			0.95			>0.99
	Day14			0.91			0.52			0.26			0.99			0.81
vit-5,-6 Kan	Day 1		>0.99	>0.99		0.48	0.99		>0.99	>0.99		0.4831	0.18		0.95	0.92
	Day 7		0.99	0.99		0.18	0.98		**0.0001**	0.95		0.8623	**0.0008**		**0.012**	0.88
	Day14		0.27	0.75		0.40	0.58		0.21	0.90		**0.0381**	0.65		0.308	**0.015**
L4440 Kan + FUDR	Day 1	>0.99		>0.99	0.90		0.67	>0.99		>0.99	**0.021**		0.89	0.92		0.91
	Day 7	0.90		>0.99	0.97		0.65	**<0.0001**		0.43	0.85		0.89	**0.0001**		0.86
	Day 14	**<0.0001**		0.32	**0.0007**		0.64	0.80		0.94	0.23		>0.99	**<0.0001**		>0.99
vit-5,-6 RNAi Kan + FUDR	Day 1	>0.99	>0.99	>0.99	0.52	0.77	0.91	>0.99	>0.99	>0.99	0.81	0.90	0.97	0.93	0.89	0.85
	Day 7	0.98	>0.99	0.99	0.87	0.27	0.99	**<0.0001**	0.38	0.99	0.49	0.44	0.49	0.94	0.99	0.80
	Dar 14	0.30	0.91	0.52	**0.016**	0.93	>0.99	0.39	**0.026**	0.85	0.55	*0.062*	>0.99	**<0.0001**	**0.0144**	0.79

Bold, $p < 0.05$; italics, $0.1 > p > 0.05$.

bioRxiv preprint doi: https://doi.org/10.1101/2025.08.21.671442; this version posted August 25, 2025. The copyright holder for this preprint (which was not certified by peer review) is the author/funder, who has granted bioRxiv a license to display the preprint in perpetuity. It is made available under aCC-BY 4.0 International license.

Table S12. Combined effects of blocking infection, tumor development and vitellogenesis (20˚C)

**Strain/ conditions**	**Number of deaths/ censored**	**Mean [Median] lifespan (days)**	**% change vs. L4440**	***p* vs. L4440 (log rank)**	**% change vs. L4440 treatment**	***p* vs. L4440 treatment (log rank)**	***p* vs. L4440 treatment (CPH test)**
L4440	**[C] 182/16**	**17.09 [17]**					
	[1] 57/5	17.53 [17]					
	[2] 56/7	17.25 [17]					
	[3] 69/4	16.59 [17]					
vit-5,-6 RNAi	**[C] 175/22**	**20.58 [21]**	**+20.42 [+23.53]**	**<0.0001**			
	[1] 57/7	20.02 [21]	+14.20 [+23.53]	0.0096			
	[2] 59/9	20.81 [21]	+20.64 [+23.53]	0.0007			
	[3] 59/6	20.88 [21]	+25.86 [+23.53]	0.0001			
L4440 FUDR	**[C] 192/7**	**17.14 [15]**	**+0.29 [-11.76]**	**0.7062**			
	[1] 62/0	17.65 [15]	+0.68 [-11.76]	0.9728			
	[2] 63/1	17.83 [18]	+3.36 [+5.88]	0.8844			
	[3] 67/6	16.03 [15]	-3.38 [-11.76]	0.5164			
vit-5,-6 RNAi	**[C] 177/5**	**21.97 [23]**	**+28.55 [+35.29]**	**<0.0001**	**+28.18 [+53.33]**	**<0.0001**	**0.0133**
FUDR	[1] 65/0	22.43 [23]	+27.95 [+35.29]	<0.0001	+27.08 [+53.33]	<0.0001	0.0363
	[2] 53/1	22.82 [23]	+32.29 [+35.29]	<0.0001	+27.99 [+27.78]	<0.0001	0.1656
	[3] 59/4	20.69 [21]	+24.71 [+23.53]	<0.0001	+29.07 [+40.00]	<0.0001	0.8582
L4440 Kan	**[C] 189/22**	**22.08 [21]**	**+29.20 [+23.53]**	**<0.0001**			
	[1] 63/8	23.84 [25]	+36.00 [+47.06]	<0.0001			
	[2] 61/9	22.28 [21]	+29.16 [+23.53]	<0.0001			
	[3] 65/5	20.20 [19]	+21.76 [+11.76]	0.0006			
vit-5,-6 RNAi	**[C] 184/17**	**23.26 [23]**	**+36.10 [+35.29]**	**<0.0001**	**+5.34 [+9.52]**	**0.0190**	**0.0659**
Kan	[1] 57/5	23.11 [23]	+31.83 [+35.29]	<0.0001	-3.06 [-8.00]	0.9192	0.0974
	[2] 63/5	22.60 [21]	+31.01 [+23.53]	<0.0001	+1.44 [0]	0.3177	0.2023
	[3] 64/7	24.03 [23]	+44.85 [+35.29]	<0.0001	+18.96 [+21.05]	0.0008	0.9379
L4440	**[C] 182/12**	**23.43 [23]**	**+37.10 [+35.29]**	**<0.0001**			
Kan + FUDR	[1] 60/3	23.82 [25]	+35.88 [+47.06]	<0.0001			
	[2] 62/3	23.15 [23]	+34.20 [+35.29]	<0.0001			
	[3] 60/6	23.35 [23]	+40.75 [+35.29]	<0.0001			
vit-5,-6 RNAi	**[C] 191/13**	**24.69 [25]**	**+44.47 [+47.06]**	**<0.0001**	**+5.38 [+8.70]**	**0.0140**	**0.0241**
Kan + FUDR	[1] 59/5	23.88 [25]	+36.22 [+47.06]	<0.0001	+0.25 [+0]	0.5270	0.5851
	[2] 71/2	25.35 [27]	+43.42 [+58.82]	<0.0001	+9.50 [+17.39]	0.0330	0.9309
	[3] 61/6	24.74 [25]	+49.13 [+47.06]	<0.0001	+5.95 [+8.70]	0.2210	0.0489

bioRxiv preprint doi: https://doi.org/10.1101/2025.08.21.671442; this version posted August 25, 2025. The copyright holder for this preprint (which was not certified by peer review) is the author/funder, who has granted bioRxiv a license to display the preprint in perpetuity. It is made available under aCC-BY 4.0 International license.

bioRxiv preprint doi: https://doi.org/10.1101/2025.08.21.671442; this version posted August 25, 2025. The copyright holder for this preprint (which was not certified by peer review) is the author/funder, who has granted bioRxiv a license to display the preprint in perpetuity. It is made available under aCC-BY 4.0 International license.

Table S13. Prevention of *E. coli* infection causes *ced-3* to shorten lifespan

**Strain/ conditions**	**Number of deaths/ censored**	**Mean [median] lifespan (days)**	**% change vs. N2 control**	**p vs. N2 (log rank)**	**% change vs. N2 Carb**	**p vs. N2 Carb (log rank)**
N2 control	**[C] 126/26**<br>[1] 46/13<br>[2] 56/4<br>[3] 23/9	**17.46 [17]**<br>16.74 [17]<br>18.62 [17]<br>16.04 [16]				
ced-3(n717)	**[C] 132/20**<br>[1] 53/7<br>[2] 56/4<br>[3] 23/9	**18.47 [18]**<br>18.52 [17]<br>18.40 [20]<br>18.60 [18]	**+5.78 [+5.88]**<br>+10.63 [0]<br>-1.18 [+17.65]<br>+15.96 [+12.50]	**0.1226**<br>0.0415<br>0.7763<br>0.1401		
ced-3(n1286)	**[C] 110/43**<br>[1] 48/12<br>[2] 41/19<br>[3] 21/12	**17.97 [18]**<br>17.41 [17]<br>18.79 [20]<br>17.62 [16]	**+2.92 [+5.88]**<br>+4.00 [0]<br>+0.91 [+17.65]<br>+9.85 [0]	**0.3327**<br>0.2474<br>0.7951<br>0.2799		
ced-3(n2454)	**[C] 103/22**<br>[1] 55/5<br>[2] 25/5<br>[3] 23/8	**18.04 [19]**<br>17.75 [19]<br>18.02 [20]<br>18.74 [18]	**+3.32 [+11.76]**<br>+6.03 [+11.76]<br>-3.22 [+17.65]<br>+16.83 [+12.50]	**0.7851**<br>0.3895<br>0.2628<br>0.1975		
N2 Carb	**[C] 167/24**<br>[1] 52/8<br>[2] 57/3<br>[3] 58/13	**26.28 [27]**<br>25.83<br>[26.5]<br>27.68 [27]<br>25.31 [25]	**+50.52 [+58.82]**<br>+54.30 [+55.88]<br>+48.66 [+58.82]<br>+57.79 [+56.25]	**<0.0001**<br><0.0001<br><0.0001<br><0.0001		
ced-3(n717) Carb	**[C] 140/41**<br>[1] 45/15<br>[2] 54/6<br>[3] 41/20	**23.42 [22]**<br>23.31 [21]<br>23.52 [22]<br>23.40 [23]	**+34.14 [+29.41]**<br>+39.25 [+23.53]<br>+26.32 [+29.41]<br>+45.89 [+43.75]	**<0.0001**<br><0.0001<br><0.0001<br><0.0001	**-10.88 [-18.52]**<br>-9.76 [-20.75]<br>-15.03 [-18.52]<br>-7.55 [-8.00]	**<0.0001**<br>0.0284<br>0.0002<br>0.0347
ced-3(n1286) Carb	**[C] 103/47**<br>[1] 39/21<br>[2] 41/19<br>[3] 23/7	**23.30 [23]**<br>22.96 [21]<br>23.97 [24]<br>22.91 [23]	**+33.45 [+35.29]**<br>+37.16 [+23.53]<br>+28.73 [+41.18]<br>+42.83 [+43.75]	**<0.0001**<br><0.0001<br><0.0001<br>0.0002	**-11.34 [-14.81]**<br>-11.11 [-20.75]<br>-13.40 [-11.11]<br>-9.48 [-8.00]	**<0.0001**<br>0.0249<br>0.0018<br>0.0361
ced-3(n2454) Carb	**[C] 127/64**<br>[1] 47/13<br>[2] 50/10<br>[3] 30/41	**22.04 [22]**<br>22.15 [21]<br>22.19 [22]<br>21.64 [21]	**+26.23 [+29.41]**<br>+32.32 [+23.53]<br>+19.17 [+29.41]<br>+34.91 [+31.25]	**<0.0001**<br><0.0001<br>0.0189<br>0.0007	**-16.13 [-18.52]**<br>-14.25 [-20.75]<br>-19.83 [-18.52]<br>-14.50 [-16.00]	**<0.0001**<br>0.0043<br><0.0001<br>0.0024

Table S14. Carb suppresses enhancement of *daf-2* longevity by *glp-4(bn2)* (25˚C from L4 stage)

Strain/ condition	Number of deaths/censored	Mean [median] lifespan (days)	% change vs. N2 control	p vs. N2 control (log rank)	% change vs. untreated	p vs. untreated (log rank)	% change vs. non *glp-4*	p vs. non *glp-4* (log rank)
N2 Control	**[C] 147/33**<br>[1] 50/10<br>[2] 48/12<br>[3] 49/11	**12.70 [13]**<br>11.31 [11]<br>14.04 [13]<br>12.83 [12]						
N2 Carb	**[C] 138/22**<br>[1] 57/3<br>[2] 35/5<br>[3] 46/14	**19.05 [19.5]**<br>19.12 [20]<br>18.20 [17]<br>19.61 [19]	**+50.00 [+50.00]**<br>+69.05 [+81.82]<br>+29.63 [+30.77]<br>+52.84 [+58.33]	**<0.0001**<br><0.0001<br>0.0018<br><0.0001	**+50.00 [+50.00]**<br>+69.05 [+81.82]<br>+29.63 [+30.77]<br>+52.84 [+58.33]	**<0.0001**<br><0.0001<br>0.0018<br><0.0001		
daf-2(m577)	**[C] 109/71**<br>[1] 47/13<br>[2] 31/29<br>[3] 31/29	**23.29 [25]**<br>25.91 [29]<br>24.46 [26]<br>18.01 [16]	**+83.39 [+92.31]**<br>+129.09 [+163.64]<br>+74.22 [+100.00]<br>+40.37 [+33.33]	**<0.0001**<br><0.0001<br><0.0001<br>0.0011				
daf-2(m577) Carb	**[C] 95/75**<br>[1] 36/24<br>[2] 25/25<br>[3] 34/26	**37.42 [39]**<br>34.58 [36]<br>37.28 [38]<br>40.53 [41]	**+194.65 [+200.00]**<br>+205.75 [+227.27]<br>+165.53 [+192.31]<br>+215.90 [+241.67]	**<0.0001**<br><0.0001<br><0.0001<br><0.0001	**+60.67 [+56.00]**<br>+33.46 [+24.14]<br>+52.41 [+46.15]<br>+125.04 [+156.25]	**<0.0001**<br>0.0018<br><0.0001<br><0.0001		
glp-4(bn2)	**[C] 174/6**<br>[1] 59/1<br>[2] 58/2<br>[3] 57/3	**12.31 [12]**<br>12.41 [13]<br>12.10 [10.5]<br>12.44 [12]	**-3.07 [-7.69]**<br>+9.73 [+18.18]<br>-13.82 [-19.23]<br>-3.04 [0]	**0.4434**<br>0.3249<br>0.0455<br>0.7646			**-3.07 [-7.69]**<br>+9.73 [+18.18]<br>-13.82 [-19.23]<br>-3.04 [0]	**0.4434**<br>0.3249<br>0.0455<br>0.7646
glp-4(bn2) Carb	**[C] 152/13**<br>[1] 55/5<br>[2] 44/6<br>[3] 53/2	**21.62 [22]**<br>22.29 [22]<br>20.80 [20]<br>21.62 [22]	**+70.24 [+69.23]**<br>+97.08 [+100.00]<br>+48.15 [+53.85]<br>+68.51 [+83.33]	**<0.0001**<br><0.0001<br><0.0001<br><0.0001	**+75.63 [+83.33]**<br>+79.61 [+69.23]<br>+71.90 [+90.48]<br>+73.79 [+83.33]	**<0.0001**<br><0.0001<br><0.0001<br><0.0001	**+13.49 [+12.82]**<br>+16.58 [+10.0]<br>+14.29 [+17.65]<br>+10.25 [+15,79]	**<0.0001**<br><0.0001<br>0.0092<br>0.0075
glp-4(bn2); daf-2(m577)	**[C] 171/4**<br>[1] 58/2<br>[2] 55/0<br>[3] 58/2	**37.51 [41]**<br>35.71 [39]<br>38.18 [41]<br>38.66 [41]	**+195.35 [+215.38]**<br>+215.74 [+254.55]<br>+171.94 [+215.38]<br>+201.33 [+241.67]	**<0.0001**<br><0.0001<br><0.0001<br><0.0001			**+61.05 [+64.00]**<br>+37.82 [+34.83]<br>+56.09 [+57.69]<br>+114.66 [+156.25]	**<0.0001**<br><0.0001<br><0.0001<br><0.0001
glp-4(bn2); daf-2(m577) Carb	**[C] 176/4**<br>[1] 60/0<br>[2] 57/3<br>[3] 59/1	**37.59 [41]**<br>39.20 [43]<br>36.56 [38]<br>36.93 [41]	**+195.98 [+215.38]**<br>+246.60 [+290.91]<br>+160.40 [+192.31]<br>+187.84 [+241.67]	**<0.0001**<br><0.0001<br><0.0001<br><0.0001	**+0.21 [0]**<br>+9.77 [+10.26]<br>-4.24 [-7.32]<br>-4.47 [0]	**0.0043**<br>0.0004<br><0.0001<br>0.7302	**+0.45 [+5.13]**<br>+13.36 [+19.44]<br>-1.93 [0]<br>-8.88 [0]	**0.0015**<br><0.0001<br>0.1670<br>0.8584

bioRxiv preprint doi: https://doi.org/10.1101/2025.08.21.671442; this version posted August 25, 2025. The copyright holder for this preprint (which was not certified by peer review) is the author/funder, who has granted bioRxiv a license to display the preprint in perpetuity. It is made available under aCC-BY 4.0 International license.

Table S15. Mortality deconvolution analysis of effects of *glp-4(bn2)* on lifespan in *daf-2(m577)* (no Carb)

Strain/ condition	Number of deaths/ censored	%P[1]	All deaths			p deaths			P deaths		
			Mean [median] lifespan (days)	% change vs. non *glp-4*	p vs. non *glp-4* (log rank)	Mean [median] lifespan (days)	% change vs. non *glp-4*	p vs. non *glp-4* (log rank)	Mean [median] lifespan (days)	% change vs. non *glp-4*	p vs. non *glp-4* (log rank)
N2	[1] 50/10	60.00	12.80 [13]			17.65 [17]			9.57 [7]		
glp-4(bn2)	[1] 57/2	56.14	12.68 [13]	-0.94 [0]	0.77	17.56 [17]	-0.51 [0]	0.68	8.88 [9]	-7.21 [+28.57]	0.52
daf-2(m577)	[1] 34/26	26.47	22.97 [31]			26.56 [31]			13.00 [13]		
glp-4(bn2); daf-2(m577)	[C] 56/4	19.64	37.34 [42]	+62.56 [+35.48]	<0.0001	42.00 [45]	+58.13 [+45.16]	<0.0001	18.27 [21]	+40.54 [+61.54]	0.29

[1]Proportion of P deaths, i.e. where necropsy showed a swollen, infected pharynx.

bioRxiv preprint doi: https://doi.org/10.1101/2025.08.21.671442; this version posted August 25, 2025. The copyright holder for this preprint (which was not certified by peer review) is the author/funder, who has granted bioRxiv a license to display the preprint in perpetuity. It is made available under aCC-BY 4.0 International license.

bioRxiv preprint doi: https://doi.org/10.1101/2025.08.21.671442; this version posted August 25, 2025. The copyright holder for this preprint (which was not certified by peer review) is the author/funder, who has granted bioRxiv a license to display the preprint in perpetuity. It is made available under aCC-BY 4.0 International license.

References

Gems, D. and Riddle, D.L., 2000. Genetic, behavioral and environmental determinants of male longevity in *Caenorhabditis elegans*. Genetics. 154, 1597-1610.

McCulloch, D. and Gems, D., 2003. Evolution of male longevity advantage in nematodes. Aging Cell. 2, 165-173.

McCulloch, D. and Gems, D., 2007. Sex-specific effects of the DAF-12 steroid receptor on aging in Caenorhabditis elegans. Ann N Y Acad Sci. 1119, 253-9.
